# Treatment of critical aluminum phosphide (rice tablet) poisoning with high-dose insulin: a case report

**DOI:** 10.1186/s13256-022-03425-4

**Published:** 2022-05-17

**Authors:** Moslem Sedaghattalab

**Affiliations:** Department of Internal Medicine, Emam Sajad Hospital, Yasuj University of Medical Sciences, Yasuj, Iran

**Keywords:** Aluminum phosphide, Rice tablet, Insulin, GIK, Case report

## Abstract

**Background:**

Aluminum phosphide (rice tablet) is a highly efficient agent for preserving grains against rodents and insects. It accounts for a large number of poisoning cases. Aluminum phosphide poisoning has a high mortality rate of about 90%, and to date, no antidote is available. It releases phosphine gas after exposure to moisture, and this reaction is catalyzed by the acidity of the stomach. Phosphine is then absorbed throughout the respiratory or gastrointestinal tracts and causes toxicity through inhibition of cytochrome *c* oxidase and formation of highly reactive free radicals. Treatment of patients with aluminum phosphide poisoning is supportive, including mechanical ventilation and vasopressors. The usage of infusion of glucose-insulin-potassium in rice tablet poisoning has been suggested, after its positive beneficial cardiac inotropic effects in patients with beta-blocker and calcium channel blocker poisoning.

**Case presentation:**

We report the case of a 30-year-old Iranian woman with critical aluminum phosphide poisoning, presented with hypotension and other signs of shock and severe metabolic acidosis, successfully treated with high-dose regular insulin and hypertonic dextrose and discharged from hospital in good condition. In contrast to our previous experiences, in which nearly all patients with critical aluminum phosphide poisoning died, this patient was saved with glucose-insulin-potassium.

**Conclusion:**

Aluminum phosphide poisoning has a high mortality rate, and to date, no antidote is available. Administration of high-dose intravenous regular insulin and dextrose is suggested as a potential life-saving treatment for patients with critical aluminum phosphide poisoning.

## Background

Agriculture is a major source of income for a high percentage of the rural population, and pesticides are the most frequent causes of poisoning among them. Aluminum phosphide (rice tablet) is a highly efficient agent for preserving grains against rodents and insects [1]. It accounts for a large number of poisoning cases in Iran [2]. Rice tablet poisoning is associated with a high mortality rate, ranging from 30% to 80%, mostly within the first 1–2 days after admission [3].

Rice tablet releases phosphine gas after exposure to moisture, and this reaction is catalyzed by the acidity of the stomach. Phosphine is then absorbed through the respiratory or gastrointestinal tracts and causes toxicity through inhibition of cytochrome *c* oxidase and formation of highly reactive free radicals [4].

Treatment of patients with aluminum phosphide poisoning is supportive, including mechanical ventilation and vasopressors, whereas to date, no antidote is available. Rice tablet poisoning has a high mortality rate, despite intensive care. Many therapeutic options have been tried, including gastrointestinal decontamination with sodium bicarbonate or coconut oil, potassium permanganate, gastric ventilation, intravenous magnesium sulfate, intravenous lipid emulsion, *N*-acetyl cysteine, extracorporeal membrane oxygenation, and whole blood exchange transfusion. However, these treatments have remained controversial, and more studies are required to confirm their efficacy [1].

The usage of infusion of GIK (glucose-insulin-potassium) in rice tablet poisoning has been suggested, after its positive beneficial cardiac inotropic effects in patients with beta-blocker and calcium channel blocker poisoning [5, 6]. Calcium channel blocker poisoning results in metabolic abnormalities including hyperglycemia, insulin deficiency, and metabolic acidosis resembling diabetic ketoacidosis. Aluminum phosphide poisoning also has similar metabolic derangements, which results in hyperglycemia and insulin resistance [1].

We present herein the case of a patient with critical aluminum phosphide poisoning who presented with hypotension, hypoxemia, and severe metabolic acidosis and was successfully treated with high-dose insulin and hypertonic dextrose and discharged from hospital in good condition.

## Case presentation

A 30-year-old Iranian woman from Yasuj, Iran presented to the emergency ward with aluminum phosphide poisoning, nausea, vomiting, and urine and stool incontinency. She also had a history of depression and irritable bowel syndrome. Medication was sertraline and sodium valporate. Her family had no history of congenital diseases. She had also no past medical history of poisoning or drug overdose.

On examination, her body temperature, blood pressure, heart rate, respiratory rate, and oxygen saturation were 36.5 °C, 90/60 mmHg, 110 beats/min, 35 breaths/min, and 88%, respectively.

The patient was ill and drowsy. All other examinations were normal. She was admitted to the intensive care unit (ICU) with signs of shock, and mechanical ventilation was started.

Hemoglobin was 12.5 (g/dL), the white blood cell count was 8000 (mm^3^), and the platelet count was 168,000 (per µL) at arrival. Aspartate aminotransferase was 27 (IU/L), alanine aminotransferase was 49 (IU/L), albumin was 2.9 (g/dL), alkaline phosphatase was 160 (IU/L), total bilirubin was 1.2 (mg/dL), conjugated bilirubin was 0.4 (mg/dL), pH (potential of hydrogen) was 7.12, CO_2_ (carbon dioxide) was 12.4 (mmHg), HCO_3_ (bicarbonate) was 3.9 (mEq/L), potassium was 3.9 (mEq/L), sodium was 142 (mEq/L), calcium was 8 (mg/dL), magnesium was 2 (mg/dL), phosphorus was 1.9 (mg/dL), blood sugar was 131 (mg/dL), creatinine was 0.5 (mg/dL), and blood urea nitrogen was 11.6 (mg/dL). Other laboratory tests, such as partial thromboplastin time, prothrombin time, and troponin, were normal.

Electrocardiogram (ECG) revealed sinus tachycardia. Computed tomography (CT) scan of chest revealed mild pleural effusion and atelectasis of left lower lobe of lung.

Treatment with hydrocortisone (100 mg doses at 8-h intervals), sodium bicarbonate (to maintain serum bicarbonate above 20 mEq/L), high-dose intravenous regular insulin (70 unit/h, calculated based on the formula 1 IU/kg/h), hypertonic dextrose 50% (1 cc/kg to maintain serum glucose around 150 mg/dL), potassium (to maintain serum potassium between 3.5 and 4.5 mEq/L), intravenous pantoprazole for stress ulcer prophylaxis, diazepam for sedation, subcutaneous heparin for deep vein thrombosis prophylaxis, normal saline for initial hydration and maintenance serum therapy, and mechanical ventilation was initiated. The signs and symptoms of shock resolved completely, and GIK was tapered within 4 days after resolution of hypotension and acidosis. Finally, the patient was discharged from hospital in good condition, satisfied with the treatment and medical team.

## Discussion

Aluminum phosphide poisoning has no specific antidote, in spite of the high mortality of about 90% in some studies, and patients generally die due to multiorgan failure [7].

Administration of GIK assists myocardial uptake of carbohydrates, which are the preferred fuel substrates of the myocard, under stressed conditions [8]. GIK was first introduced as a possible treatment for rice tablet poisoning in 2008, in a small number of patients [9].

Insulin infusion improves inotropy of heart and peripheral vascular resistance and reverses acidosis, by increasing myocyte carbohydrate uptake and utilization. In addition, GIK therapy may increase the metabolism of lactate and limit the metabolic acidosis commonly seen in rice tablet poisoning [10].

In a study by Hassanian-Moghaddam *et al.* on 88 aluminum phosphide poisoning patients, 44 of them receiving insulin infusion, which supports our experience, administering GIK (loading dose of regular insulin set at 1–3 IU/kg/h, followed by 0.2–0.5 IU/kg/h with glucose and potassium to maintain serum glucose around 150 mg/dL and serum potassium between 3.5 and 4 mEq/L) had significant effect on improving survival rate and decreasing death cases, although their study has some limitations, such as low sample size and nonblinded randomization. In addition, other clinically relevant parameters, such as frequency or duration of mechanical ventilation, and dose of vasoactive support, were not investigated at all [7].

In another pilot study by Pannu *et al.* on 60 rice tablet poisoning patients, 30 of them receiving insulin infusion (insulin regular 0.1–0.5 IU/kg/h with glucose and potassium to maintain serum glucose and serum potassium between 150 and 200 mg/dL and 3.5–4.5 mEq/L, respectively), the survival rate was significantly higher in the GIK group, although they also had a small sample size and power, and their study was unblinded [1].

We describe herein the case of a critical rice tablet poisoning patient who presented with hypotension and other signs of shock and severe metabolic acidosis. She had metabolic acidosis due to inhibition of cytochrome *C* oxidase and formation of highly reactive free radicals, caused by phosphine. Her hypotension was due to negative inotropic effects of acidosis and phosphine on myocardium.

In contrast to our previous experiences, in which nearly all aluminum phosphide poisoning patients with hypotension and severe metabolic acidosis died, this patient miraculously was saved with GIK. Treatment with hydrocortisone, high-dose intravenous regular insulin, hypertonic dextrose 50%, potassium, and mechanical ventilation was initiated; the signs and symptoms of shock resolved completely, and the patient was discharged from hospital in good condition.

## Conclusion

Aluminum phosphide poisoning has a high mortality rate of about 90%, and to date, no antidote is available. We describe herein a patient with critical aluminum phosphide poisoning who was successfully treated with high-dose insulin and hypertonic dextrose, and discharged from hospital in good condition. As a result, high-dose intravenous regular insulin and dextrose is suggested as a potential life-saving treatment for critical aluminum phosphide poisoning patients.

## Data Availability

The datasets of the current case presentation are available from the corresponding author on reasonable request.

## Abbreviations

GIK: Glucose-insulin-potassium
ICU: Intensive care unit
ECG: Electrocardiogram
CT: Computed tomography
